# Evaluations of Factors Predicting the Need for an Extra-Cervical Approach for Intra-Thoracic Goiter

**Published:** 2015-11

**Authors:** Ali Sadrizadeh, Sadeq Ghafarian, Seyed Ziaollah Haghi, Maryam Salehi

**Affiliations:** 1 *Cardiothoracic Surgery & Transplant Research Center, Emam Reza Hospital, Faculty of Medicine, Mashhad University of Medical Sciences, Mashhad, Iran.*; 2 *Department of Community Medicine, Faculty of Medicine, Mashhad University of Medical Sciences, Mashhad, Iran. *

**Keywords:** Cervical incision, Intra-thoracic goiter, Mediastinal goiter, Sub-sternal goiter, Sternotomy, Thyroid, Thyroidectomy

## Abstract

**Introduction:**

Intra-thoracic goiter refers to the extension of enlarged thyroid tissue into the thoracic inlet. This condition can produce symptoms of compression on adjacent organs and can sometimes be accompanied by malignant transformation. Therefore surgical treatment is almost always necessary. In order to remove the pathology with the fewest post-operative complications, selection of the appropriate surgical approach is essential. In this study we aimed to detect the criteria which help us select the best therapeutic approach.

**Materials and Methods:**

In this retrospective study, 82 patients with intra-thoracic goiter were investigated. Their data were extracted from medical records and analyzed using SPSS software.

**Results:**

Overall 82 patients, 18 (21%) males and 64 (78%) females with mean age of 56.38 years were studied. The most common clinical symptoms were mass (95%) and dyspnea (73%). In most patients, the surgical approach was cervical (90.2%), while 9.8% of patients required an extra-cervical approach. Post-operation complications were observed in 17.1% of patients; the most common being transient recurrent laryngeal nerve paralysis (4.9%). Malignancy was reported in the histopathology of seven patients (8.5%). The most common malignant histopathology was papillary thyroid carcinoma (7.3%). Extension of the thyroid tissue below the uppermost level of the aortic arch was significantly correlated with the need for an extra-cervical approach to surgery (P<0.001).

**Conclusion:**

Because of the compressive effect and risk of malignancy, intra-thoracic goiters require immediate surgical intervention. Commonly, cervical incision is used for removing the extended goiter to the mediastinum. Extension of the goiter below the uppermost level of the aortic arch increases the likelihood of an extra-cervical approach being required.

## Introduction

Intra-thoracic goiter was first described by Haller in 1749, as a condition in which the thyroid gland is located in the thorax. Other terms such as retrosternal, sub-sternal, subclavicular and intra-thoracic goiter are also used to describe the subtypes of the condition (1,2). There is currently no consensus on the definition of these clinical and pathological analogies, and no agreement on how much of the thyroid tissue should be under the thoracic inlet to diagnose intra-thoracic goiter (2). The definition offered by Katlic seems the most appropriate, in which an intra-thoracic goiter is diagnosed when 50% of the thyroid mass is extended downwards to the thoracic inlet (3). Primary mediastinal goiter occurs in 1% of intra-thoracic goiters, and is caused by abnormal growth of the embryonic thyroid cells into the thoracic inlet and is usually isolated from the main thyroid mass in the neck. Acquired retrosternal goiter is more common, in which the main tissue of the cervical thyroid gland grows as much as to descend into the thorax (1,4,5). A computed tomography (CT) scan is the most useful imaging modality for determining the relationship between the goiter and other organs in the thorax and to decide on the best surgical approach (6). As previously described, an intra-thoracic goiter causes a compressive effect on the adjacent organs; therefore, surgery is inevitable (7). 

In most patients, cervical incision is sufficient to remove the enlarged gland, but some cases may require sternotomy or thoracotomy. Determining the most suitable method to eliminate all pathology and to minimize post-operative complications is very important (5,8). In this study we aimed to identify the criteria which help select the best surgical approach.

## Materials and Methods

In this study, 82 patients with an intra-thoracic goiter undergoing surgery in our teaching hospital from 2004–2013 were investigated. After clinical examination, patients were referred for a chest X-ray, and when intra-thoracic goiter was suspected, patients were referred for a subsequent diagnostic CT scan.

We reviewed the CT scans of patients and measured the uppermost to lowermost dimension of the goiter using the sternal notch as an indicator for the separation of the neck from the mediastinum. Because a change in neck position can alter this calculation, all CT scans were performed in the same position. Diagnosis of intra-thoracic goiter was confirmed when either more than 50% of the thyroid gland was located in the mediastinum or the goiter reached the uppermost level of the aortic arch, regardless of the percentage of intra- or extra-thoracic component. In patients with an intra-thoracic goiter, compression symptoms and cervical large mass were indications for surgery. For all patients, cervical incision was performed initially; however before surgery, the necessity of an extra-cervical approach was considered based on the extension of the mediastinal thyroid and CT scan imaging. However, the final decision was made in the operating room. Surgery was started by cervical incision and, after exposing the thyroid, the median thyroid vein and the superior thyroid vessels were ligated and the parathyroid glands were maintained. Typically, it was not possible to localize the inferior thyroid artery or recurrent laryngeal nerve prior to removal of the intra-thoracic section. Therefore, initial attempts were made to remove the intra-thoracic part after releasing it by finger from the thyroid capsule, while at the same time ensuring that the recurrent laryngeal nerve and parathyroid glands were not injured. Whenever it was possible to remove the thyroid via a cervical approach, this was done. Otherwise, another approach was selected based on mediastinal thyroid and the positioning of the mediastinal great vessels. Based on CT scan imaging, if the mediastinal part was located in front of the great vessels, we performed limited median sternotomy, while if it was located at the posterior of the great vessels, a third-space limited anterior thoracotomy was performed. The patients were followed up 2,6,12, and 24 months after surgery, and thyroid hormones and calcium levels were investigated at these intervals. In addition to clinical examinations, special complications such as dysphagia and odynophagia were sought. 

The study was performed in a retrospective manner and the information collated was extracted from clinical files. This information consisted of age, gender, surgical approach, surgical complications, and goiter histopathology, as evaluated in all patients. Other information such as the position of the goiter in the mediastinum, previous history of thyroid operation, follow-up duration and admission period were also considered.

*Statistical analysis*

Descriptive data were analyzed using SPSS 11.5. Data with normal distribution were presented as mean±standard deviation (SD). An independent sample t-test was used for quantitative variables and χ^2^ and Fisher exact tests were used for qualitative variables. A p-value less than 0.05 was considered significant. 

## Results

Overall 82 patients (64 females and 18 males; mean age, 56.38 years; range, 24–81 years) were enrolled in this study. The mean age of the male participants was 63.89 years compared with 54.27 years for females (P=0.007).

Most patients were aged between 61–70 years (28%). Mass (95.1%) and dyspnea (73.2%) were the most common clinical symptoms among patients. Two cases presented with no symptoms and were identified during other health assessments. In one of these cases, this was due to a life-threatening compressive effect on the trachea, requiring the patient to be immediately intubated in the emergency room and undergo emergency surgery. Also due to tracheomalacia, this patient was re-intubated for a limited time following surgery (Table.1).

**Table 1:** Incidence of clinical symptoms in intra-thoracic goiter patients

**Clinical symptoms**	**Number of patient**	**Incidence of symptoms (percent)**
Mass	78	95.1
Shortness of breath (dyspnea)	60	73.2
Dysphagia	7	8.5
Dysphonia	2	2.4
Vena cava syndrome	1	1.2
Pain in chest	1	1.2
Asymptomatic	2	2

In most patients (90.2%), surgery was performed using a cervicotomy approach, but in 9.8% cases, an extra-cervical approach was used (Table.2).

**Table 2:** Frequency of surgical approach in the patients

**Surgical approach**	**Number (%)**
Cervicotomy	74 (90.2)
Sternotomy and cervicotomy	6 (7.3)
Thoracotomy and cervicotomy	2 (2.4)

Mean ± SD and mode for admission time after the operation was 2.52 ± 1.2 and 2 days, and follow-up rates were 31.8 ± 26.27 and 24 months, respectively. In our study, 68 patients had no post-operative complications but in 14 patients (17.1%) some complications were reported. The most common complications were transient recurrent laryngeal nerve paralysis that recovered after 6 months (Table.3). 

**Table 3:** Post-operative complications of patients undergoing surgery

**Post-operative complications**	**Number of patients**	**Percentage of patients**
No complications	68	82.9
Transient recurrent laryngeal nerve paralysis	4	4.9
Permanent recurrent laryngeal nerve paralysis	3	3.7
Temporary hypoparathyroidism	3	3.7
Permanent hypoparathyroidism	1	1.2
Transient phrenic nerve paralysis	1	1.2
Tracheomalacia	1	1.2
Seroma	1	1.2

In 71 cases, information about the position of the goiter within the mediastinum was collected. The most common site was anterior goiter, in 58 cases (81.7%). The status of the enlarged gland to the aortic arch was another factor investigated in this study; in 10 patients the goiter extended below the uppermost level of the aortic arc. The reported histopathology was benign in 75 patients and malignant in seven cases (8.5%). Papillary thyroid carcinoma was the most common histology among patients (7.3%). History of previous thyroid surgery was investigated in 71 patients based on medical records, and 11 patients reported previous thyroid surgery with a mean and median of time after operation of 21.5 and 20 years (range, 2–40 years), respectively. 

In an inferential statistical analysis, the effect of age, posterior goiter extension, extension of goiter below the uppermost level of the aortic arch and histopathology were determined as risk factors for sternotomy. The surgical approach was variable and could be divided into cervical and extra-cervical approaches. Extra-cervical approaches could further be divided into sternotomy and thoracotomy subtypes. There was no significant difference in the effect of age on the surgical approach (56.49 years in the cervical group vs. 38.55 years for the extra-cervical approach; P=0.826).

For posterior goiter extension, 100% of the mass with posterior extension was removed via a cervical approach, and neither required an extra-cervical approach. This rate changed according to position of the goiter, to 89.7% (cervical) and 10.3% (extra-cervical); however, according to the Fisher exact test, this difference was not statistically significant (P= 0.65).

Concerning the extension of the goiter below the uppermost level of the aortic arch and its effect on the surgical approach, 100% of the mass with no extension below the uppermost level of the aortic arch was removed via a cervical approach, and no patients required an extra-cervical approach. This rate changed to 40% and 60%, respectively, in cases in which the goiter extended below the uppermost level of the aortic arch. The Fisher exact test revealed this difference to be statistically significant (P<0.001) (Table. 4).

**Table 4:** Effect of transmission of goiter from aortic arch on surgical approach

**Statue**	**Cervical**	**Extra-cervical**	**P-value**
Non transmission from aortic arch	100%	0%	<0.001
Transmission from aortic arch	40%	60%	<0.001

Histology variables were divided into benign and malignant for investigation of the final pathology according to surgical approach. Inferential analysis showed that 90.7% of benign masses were removed via a cervical approach and just 9.3% were removed via an extra-cervical approach. In malignant masses, these rates changed to 85.7% and 14.3%, respectively. The Fisher exact test revealed no significant difference in the comparison of these groups (P=0.52). In order to study the effect of age on post-operative complications, the patients were divided into two groups; the mean age in cases with or without post-operative complications was 56.64 % and 56.32 %, respectively. (P=0.936). 

Comparing the effect of gender, surgical approach and goiter histopathology on post-operative complications, no correlation was identified (Table. 5).

**Table 5:** Effect of gender, surgical approach and goiter histopathology on post-operative complication

	**Patients with post-operative complications**	**Patients without post-operative complications**	**P-value**
Male Female	4(22.2%) 10(15.6%)	14(77.8%) 54(84.4%)	0.49
Cervical incision Extra-cervical operation	11(14.9%) 3(37.5%)	63(85.1%) 5(62.5%)	0.13
Benign Malignant	12 (16%) 2(28.6%)	63(84%) 5(71.4%)	0.34

## Discussion

The management and treatment of intra-thoracic goiter have been areas of controversy since 1949. Several descriptions and classifications have been suggested for this clinical status, but still there is no consensus concerning the best surgical approach (9). The presence of 50% of goiter mass below the sternum as a basis for description of intra-thoracic goiter was first used by Katlic. Based on Rios’s study, this approach is valuable in predicting the need for sternotomy. In 2010, Rios’s team showed that the different descriptions for this clinical condition have led to differences in incidence reports, varying between 2.8–48% (3). In 2011, Raffaelli and colleagues used the same description for intra-thoracic goiter and showed that the incidence of intra-thoracic goiter in their department was 15.7% of thyroidectomy cases (4). In our study, the most common sign/symptom was goiter mass (95.1%) followed by dyspnea (73.2%). Our findings were similar to the study of Nistor et al. (2014) who showed that dyspnea was the most common symptom, followed by dysphagia and dysphonia (9). In our study, the most commonly used operative approach for intra-thoracic goiter was cervical incision. Overall, 90.2% of patients (74 patients) underwent a cervical surgical approach and just 9.8% (eight patients) had an extra-cervical approach. Sternotomy and cervicotomy were performed in 7.3% of these cases (six patients), and 2.4% of these cases were operated on via thoracotomy and cervicotomy. Other studies similarly reported cervicotomy in 90% percent of their surgical cases (4,7,9-11). 

We found that neither the location of the goiter in the posterior mediastinum nor the histopathology of the goiter provided sufficient data to determine the need for an extra-cervical approach; but extension of the goiter below the uppermost level of the aortic arch was significantly associated with the need for an extra-cervical approach. In 2008, Huins et al. similarly reported that goiters reaching the arc of aorta were associated with a 10-fold increase in rate of complications and the need for an extra-cervical approach (2). Cichon et al*. *reported in 2008 that recurrent goiter, primary mediastinal and posterior mediastinal location of thyroid tissue, and aberrant adenoma in the mediastinum are risk factors for sternotomy (5). In another study from Rugiu in 2009, it was concluded that the presence of an ectopic goiter, the thyroid gland volume and extension of the goiter into or below the tracheal carina were the most significant indications for sternotomy (14). The incidence of post-operative complications is variable in the literature. In some studies, such as that reported by Nistor, there was no report of mortality or vocal cord paralysis (9), while in the study by Landerholm, three mortalities were reported (13). In the study by Abboud, five patients had permanent vocal cord paralysis (12). In our study, up to 68 of 82 patients (82.9%) reported no post-operative complications and 17.1% had some complications that were mostly transient recurrent laryngeal nerve paralysis, almost all of which recovered within 6 months. 

There were no cases of mortality among our subjects.

The effect of variables such as age, gender, surgical approach and histopathology on post-operative compli- cations was investigated, and found not to be significant. We observed that the histopathology of the goiter in 75 (91.5%) and seven (8.5%) patients was benign and malignant, respectively.

Papillary thyroid carcinoma was the most common malignant histology in this study (7.3%). In the study by Rugiu in 52 subjects overall, 50 were benign and two were malignant (14). Haj-hosseini and colleagues reported an incidence of malignancy of 12.7% (10).

## Conclusion

Overall, our results confirm that due to the compressive effects and risk of malignancy, a surgical approach is necessary in the management of intra-thoracic goiter. A cervical approach can be performed in the majority of the patients, and an extra-cervical approach is not normally necessary. Post-operative complications are not very common and most are reversible. Extension of the goiter bellow the uppermost level of the aortic arch increases the likelihood of an extra-cervical approach being required. Neck and thorax CT scans are necessary prior to surgery. Further studies with more cases would be useful to understand the best approach to treatment of intra-thoracic goiter.
